# Effect of Sirolimus on the Level of Peripheral Blood Lymphocyte Autophagy in Children With Systemic Lupus Erythematosus

**DOI:** 10.3389/fped.2021.685497

**Published:** 2021-10-15

**Authors:** Xinliang Wang, Qingxiao Su, Zhiyan Dou, Xue Zhao, Naiqi Zhang, Bo Yu, Yuxue Wang, Zanhua Rong

**Affiliations:** Department of Peadiatrics, The Second Hospital of HeBei Medical University, Shijiazhuang, China

**Keywords:** sirolimus, systemic lupus erythematosus, peripheral blood lymphocyte, autophagy, children

## Abstract

**Background:** To observe the changes of autophagy-related protein levels in peripheral blood lymphocytes before and after sirolimus treatment in children with systemic lupus erythematosus (SLE).

**Methods:** Children with SLE were randomly divided into two groups, 28 in the traditional treatment group and 28 in the sirolimus group. Fifteen healthy children who were in the same period were collected as the normal control group. Clinical laboratory indexes, the percentage of routine lymphocytes, complement C3, complement C4, serum Anti-dsDNA and SLEDAI were detected.

**Results:** At 3 and 6 months after treatment, compared with the traditional treatment group, the percentage of routine lymphocytes in the sirolimus group increased (*P* = 0.03), SLEDAI score and positive rate of Anti-dsDNA decreased (*P* = 0.01). Compared with normal children, the expression of microtubule-associated protein 1 light chain 3 (LC3) protein in peripheral blood lymphocytes was significantly higher (*P* = 0.006); peripheral blood expression of P62/SQSTM1 (sequestosome 1) protein in lymphocytes decreased (*P* = 0.02).

**Conclusion:** Sirolimus can play a role in the treatment of systemic lupus erythematosus by regulating the level of autophagy.

## Introduction

Systemic lupus erythematosus (SLE) is an autoimmune disease with disordered immune system regulation. Autophagy refers to the process of using lysosomes to remove or reuse damaged organelles and metabolites in the cell under the conditions of starvation, hypoxia, infection, stress, etc. to help the cell maintain an effective energy cycle (1–5).

SLE patients have autophagy in the peripheral blood mononuclear cells, and the expression of Beclin-1 and LC3 mRNA is significantly higher than normal (6, 7). LC3 and p62/SQSTM1 (sequestosome 1) are often used as markers to detect autophagy. The study found that autophagy is associated with B lymphocytes in SLE patients. After comparing SLE patients with normal people, it was found that autophagy-related markers in B lymphocytes increased significantly. In the early stage of the onset of SLE, the LC3 expression level of lymphocytes was significantly increased, the autophagy of B lymphocytes was excessively activated, a large number of autophagosomes appeared, and there was a positive correlation change with the disease activity of SLE (8). At the same time, the number of autophagosomes in T lymphocytes of SLE patients was also found to be higher than that of normal people, indicating that the upregulation of autophagy may be involved in the process of T lymphocyte differentiation, survival and function. Therefore, it is speculated that the incidence of systemic lupus erythematosus is related to the abnormality of lymphocyte autophagy.

Sirolimus is a mammalian target of rapamycin (mTOR) inhibitor, which exerts immunosuppressive function by acting on the mTOR pathway. The mTOR pathway plays a key role in inducing autophagy (9, 10). With the continuous understanding of the pathogenesis of rheumatic immune diseases and the mechanism of sirolimus, sirolimus, as a new, powerful and low-toxic macrolide immunosuppressive drug, is gradually recognized and familiarized by clinicians. And more and more are used in clinical treatment of rheumatic immune diseases. This experiment explores the relationship between autophagy and the onset of SLE, hoping to bring new ideas to the diagnosis and treatment of SLE.

## Methods

### Materials

#### Clinical Data

The data is collected from children diagnosed with SLE (56 cases) who were admitted to the pediatric outpatient and inpatient of the Second Hospital of Hebei Medical University from December 2016 to June 2018, and the diagnosis was in accordance with the SLE classification standard (11) revised by the American College of Rheumatology (ACR) in 1997 or the SLE classification standard revised by the ACR/Systemic Lupus International Collaborating Clinic (SLICC) in 2009 (12). Children with SLE who were not treated with sirolimus, hormones or other immunosuppressive agents before enrollment were randomly divided into two groups, the traditional treatment group and the combined sirolimus group (hereinafter referred to as sirolimus group), with 28 cases in each group. The traditional treatment group was composed of 3 males and 25 females with an average age of 13 ± 1.5 years (range 10–15), and the sirolimus group was composed of 2 males and 26 females with an average age of 13.4 ± 1.4 years (range 11–16). And 15 healthy children who were treated in the growth and development clinic of our hospital during the same period were selected as the normal control group, with an average age of 11.8 ± 2.4 years (range 8–15, male/female:2/13), as shown in Table 1. Clinical data and experimental data of children with SLE before and after treatment were collected and the SLEDAI score was calculated according to the systemic lupus erythematosus disease activity index (SLEDAI) (13).

**Table 1:** Patients' characteristics.

**Factor**	**Traditional treatment** **(*n* = 28)**	**Sirolimus** **(*n* = 28)**	**Normal control** **(*n* = 15)**
Recipient age (*y*)	13 ± 1.5	13.4 ± 1.4	11.8 ± 2.4
Gender (male:female)	3:25	2:26	2:13
Fever	19	20	–
Asthenia	5	4	–
weight loss	4	3	–
Hypertension	8	11	–
Rash	24	23	–
light sensitivity	10	12	–
Mouth ulcers	8	7	–
Alopecia	11	9	–
24 h urinary protein ≥ 0.5 g/24 h	20	19	–
White blood cell count <4.0 × 10^9^/L	9	10	–
Platelet count <100 × 10^9^/L	6	8	–
Hemoglobin <100 g/L	7	9	–
IgG (g/L) quartile	19 [14.25–25.75]	18 [14.25–24.75]	–
Anti-nuclear antibody	28	28	–

#### Randomization

Randomization will be performed by an independent statistician using a randomization assignment procedure to generate a randomization sequence and randomly assign patients into two groups in a 1:1 ratio. The participant assignment table will be kept by an independent statistician until the end of the study.

#### Grouping Standards

##### Test Group

1) Twenty Eight cases in the traditional treatment group (Group A): oral prednisone 1 mg/kg/d, mycophenolate mofetil 60 0mg/m^2^/bid, and the dosage was adjusted in the follow-up clinic.
2) Twenty Eight cases in the sirolimus group (Group B), oral drug: sirolimus capsule (specification 0.5 mg/capsule, manufacturer: North China Pharmaceutical Co., Ltd., approval number: National Pharmaceutical Standard H20100079) 1 mg/m^2^/d, at the same time oral prednisone 1 mg/kg/d, mycophenolate mofetil 600 mg/m^2^/bid, and the dosage was adjusted in the follow-up clinic.

##### Normal Control Group (Group C)

Fifteen healthy children who were treated in the growth and development clinic of our hospital during the same period were selected as the control group. Medical ethics were observed and permission from the children's guardians was obtained.

### Exclusion Criteria

1) At the same time suffering from other autoimmune diseases, such as Sjogren syndrome, juvenile idiopathic arthritis, autoimmune liver disease, etc.;
2) At the same time suffering from other chronic diseases, such as parathyroid disease, diabetes, hypertension, acute and chronic infections or tumors;
3) Receive sirolimus, hormone and immunosuppressive therapy at least 6 months before enrollment;
4) Allergic reactions to the therapeutic drugs involved in the experiment.

### Collection and Collation of Clinical Data

The collected contents include percentage of blood routine lymphocytes, complement C3, complement C4, serum Anti-dsDNA, triglyceride, total cholesterol and disease activity assessment (SLEDAI) of the enrolled children. After obtaining the children's guardians' informed consent, fill it out in the specified uniform format.

### Experimental Reagents

Human peripheral blood lymphocyte separation solution (Tianjin Haoyang Biological Products Technology Co., Ltd.), Coomassie Brilliant Blue Protein Analysis Kit (Nanjing Jiancheng Bioengineering Research Institute), horseradish enzyme-labeled goat anti-rabbit IgG (imported packaging by Beijing Zhongshan Jinqiao Company), DAB coloring kit (imported packaging by Beijing Zhongshan Jinqiao Company), pre-stained protein molecular weight markers (American Thermo Scientific Company), PVDF membrane (Millipore Inc. USA), ECL enhanced chemiluminescence kit (Beijing TIANGEN Company), rabbit anti-actin polyclonal antibody (Proteintech, USA).

### Experimental Equipment

DZ5-WS centrifuge (Shanghai Luxiangyi Centrifuge Instrument Co., Ltd.), high-pressure desktop sterilizer (Sai Strontium Krypton Shanghai Trading Co., Ltd.), fluorescence microscope (Beijing Shangguang Instrument Co., Ltd.), ultra-low temperature refrigerator (Qingdao Haier), vertical electrophoresis tank (Bio-Rad, USA), transfer electrophoresis (Bio-Rad, USA), transfer electrophoresis tank (Bio-Rad, USA), Odyssey FC imager (Li-COR Biosciences, USA), pressure steam sterilizer (Medical Equipment Factory of Boxun Industrial Co., Ltd.).

## Research Methods

### Observation Indicators

Western blotting was used to detect the expression of LC3 and P62 in peripheral blood lymphocytes of children with SLE. Immunofluorescence was used to detect the expression of LC3 and P62.

### Specimen Collection and Preparation

Peripheral blood lymphocyte protein collection: Collect 5 ml of fasting peripheral blood (venous blood) from the morning, use EDTA for anticoagulation, and isolate lymphocytes within 3 h by “human peripheral blood lymphocyte separation fluid.” Add 500 ul of cell lysate to a 15 ml centrifuge tube containing lymphocytes, place it in a refrigerator at 4°C or on ice for 30 min, then transfer the supernatant to an EP tube and centrifuge (4°C, 12,000 rpm, 20 min) to extract the supernatant and obtain cellular proteins. Place the collected cellular proteins in the refrigerator at −80°C for later use. Or add a loading buffer to the protein sample solution according to the ratio of 100 ul lysate plus 20 μl 6X loading buffer, and denature the protein sample at high temperature (95°C, 5 min) and store at −20°C for later use.

### Detection Methods

#### Human Peripheral Blood Lymphocyte Isolation

Use “Human Peripheral Blood Lymphocyte Separation Solution” to separate lymphocytes and follow the steps in the “Human Peripheral Blood Lymphocyte Separation Solution Instructions” (Tianjin Haoyang Biological Products Technology Co., Ltd.). The operation is carried out under the condition of 18–22°C.

#### Coomassie Brilliant Blue Reagent Method for Protein Quantification

Set blank tube, standard tube and sample tube in the experiment. Add 3 ml of Coomassie Blue Diluent to each tube, mix, let stand for 10 min, and then test under an ultraviolet spectrophotometer. The blank tube is reset to zero, and the absorbance value of the standard tube and the sample of each tube is measured separately. The protein sample concentration (mg/ml) = sample absorbance value/standard protein absorbance value × standard protein concentration.

#### Immunoblotting to Detect Protein Expression Levels

Primary antibodies include LC3 (1:1,000 dilution in 1% BSA), P62 (1:1,000 dilution in 1% BSA), β-actin (1:1,000 dilution in 1% BSA). The secondary antibody is horseradish peroxidase-labeled goat anti-rabbit or mouse IgG secondary antibody (diluted 1:5,000 in 1% BSA solution).

### Cell Immunofluorescence Steps

1) Centrifuge the collected lymphocytes and add them to the slides and fix with 4% paraformaldehyde for 30 min.
2) Wash with PBS, 5 min/time, 3 times, Triton-100 (1:100) perforated, room temperature for 30 min.
3) Wash with PBS, 5 min/time, 3 times, block goat serum and incubate at 37°C for 30 min.
4) Add primary antibody and overnight at 4°C.
5) Re-warm for 30 min the next day, wash with PBS, 5 min/time, wash 3 times.
6) Add biomarker goat anti-rabbit IgG secondary antibody to the membrane and incubate at 37°C for 2 h.
7) Wash with PBS, 5 min/time, 3 times, stain with DAPI and seal for 30 min.
8) Observe and photograph the fluorescence result under a fluorescence microscope.

#### Formation of Autophagy Using GFP-LC3 Fusion Protein and GFP-P62 Fusion Protein Under the Fluorescent Microscope

The observation of the formation of autophagosomes under an electron microscope takes a long time, which is not conducive to the monitoring of autophagy. LC3 is a homolog of the yeast autophagy gene (Atg8) in mammals. People developed this technology by making use of the aggregation phenomenon that occurred during the autophagy of microtubule-associated protein 1 light chain 3 (LC3). This technology can be combined with a green fluorescent protein (GFP) to form GFP-LC3 to detect autophagosomes. When there is no autophagy, the GFP-LC3 fusion protein is dispersed in the cytoplasm; when autophagy occurs, the GFP-LC3 fusion protein is translocated to the autophagosome membrane, and multiple bright green fluorescent spots (puneta) are formed under a fluorescent microscope. A fluorescent spot is equivalent to an autophagosome, and the activity of autophagy can be evaluated by counting the fluorescent spots. P62 protein accumulates in the cytoplasm when autophagy is deficient, and is transferred to lysosomes for degradation when autophagy is activated. Therefore, the autophagy activity can be evaluated by counting the GFP-P62 in the cytoplasm.

## Statistical Processing

SPSS22.0 statistical software was used for statistical processing. The normal distribution data was represented by mean ± standard deviation ($\bar{x}$ ± *s*). The comparison of the mean was by one-way analysis of variance and LSD-t (Least Significant Different-t) test; A repeated measurement analysis of variance was performed to compare the differences in clinical laboratory indicators at different time points before, during and after the interventions. the comparison between groups was by Mann-Whitney Test and count data using χ^2^ test. *P* < 0.05 indicated statistical significance, and *P* < 0.01 were considered highly significant.

## Results

### Changes of Clinical Laboratory Indexes in Children With Systemic Lupus Erythematosus

1) Compared with the traditional treatment group, the percentage of blood routine lymphocytes increased at the end of 3 months of treatment in the sirolimus group, which was statistically significant (*P* = 0.03). The percentage of blood routine lymphocytes increased at the end of 6 months of treatment, and the difference was statistically significant (*P* = 0.002) (see Table 2).
2) Compared with the traditional treatment group, the sirolimus group had a decrease in SLEDAI score at the end of 3 months of treatment, and the difference was statistically significant (*P* = 0.01). At the end of 6 months of treatment, the SLEDAI scores decreased, and the difference was statistically significant (*P* = 0.001). And the positive rate of Anti-dsDNA decreased at the end of 3 and 6 months of treatment, which was statistically significant compared with the traditional treatment group (*P* = 0.02) (see Table 2).
3) Comparing the sirolimus group with the traditional treatment group, complement C3, complement C4 triglyceride, and total cholesterol increased at the end of 3 months of treatment, and the difference was not statistically significant (*P* = 0.08). At the end of 6 months of treatment, complement C3, complement C4, triglyceride, and total cholesterol were increased, and the difference was not statistically significant (*P* = 0.1). (see Table 2).
4) Compared with before treatment, experimental indicators such as 24 h urinary protein ≥ 0.5 g/24 h and White blood cell count <4.0 × 109/L in both groups were improved after treatment (see Table 2). As for side effects, one child in the Sirolimus group had transient nausea and abdominal pain on the 2nd day after oral administration.

**Table 2:** Changes in clinical laboratory indicators at the end of 3 and 6 months in the traditional treatment group and sirolimus group.

	**Traditional treatment group (*****n*** **=** **28)**			**Sirolimus group (*****n*** **=** **28)**		
	**0 month**	**3 months**	**6 months**	**0 month**	**3 months**	**6 months**
Percentage of lymphocytes	13.91 ± 3.37	21.46 ± 2.64*	23.40 ± 4.46	13.97 ± 3.49	26.41 ± 3.58^*#^	41.36 ± 6.27^#^
SLEDAI	11.26 ± 3.49	9.46 ± 1.92*	4.53 ± 2.32	11.33 ± 2.46	6.26 ± 2.25^*#^	1.73 ± 1.48^#^
negative rate of anti-dsDNA	0 (0%)	4 (14.2%)	9 (32.1%)	0 (0%)	10 (36%)^#^	22 (78.6%)^#^
Positive rate of anti-dsDNA	28 (100%)	24 (85.8%)	19 (67.9%)	28 (100%)	18 (64.3%)^#^	6 (21.4%)^#^
Complement C3	0.62 ± 0.13	0.99 ± 0.15*	1.23 ± 0.10	0.66 ± 0.13	1.02 ± 0.19*	1.24 ± 0.22
Complement C4	0.08 ± 0.03	0.18 ± 0.04*	0.20 ± 0.05	0.10 ± 0.03	0.19 ± 0.05*	0.21 ± 0.06
Triglycerides	1.93 ± 0.63	2.03 ± 0.56*	2.94 ± 0.96	1.86 ± 0.66	2.41 ± 0.94*	2.99 ± 1.01
Total cholesterol	4.08 ± 1.22	4.87 ± 1.79*	4.99 ± 1.72	3.99 ± 1.03	4.52 ± 1.44*	5.17 ± 1.89
24 h urinary protein ≥ 0.5 g/24 h	20	0	0	19	0	0
White blood cell count <4.0 × 10^9^/L	9	4	1	10	5	2
Platelet count <100 × 10^9^/L	6	0	0	8	0	0
Hemoglobin <100 g/L	7	1	0	9	3	1
IgG (g/L) quartile	20 [17–28]	14 [10–16]	9.9 [8.55–10.85]	18 [17–25]	16 [14–19]	14 [9.325–17.00]
Anti-nuclear antibody	28	26	25	28	25	25

Compared with SLE before treatment, *P < 0.05; Compared with the traditional treatment group, ^#^P < 0.05.

### Changes in Autophagy-Related Protein Levels

**Figure 1:**
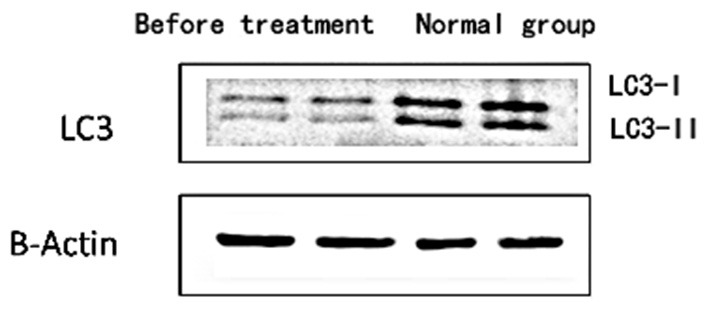
The expression level of LC3 protein in peripheral blood lymphocytes of children with systemic lupus erythematosus and normal healthy children. Protein levels of LC3 I and LC3 II were detected by western blot analysis. Two columns on the left: before treatment; two columns on the right: normal children, β-Actin was used as the internal control for normalization.

**Figure 2:**
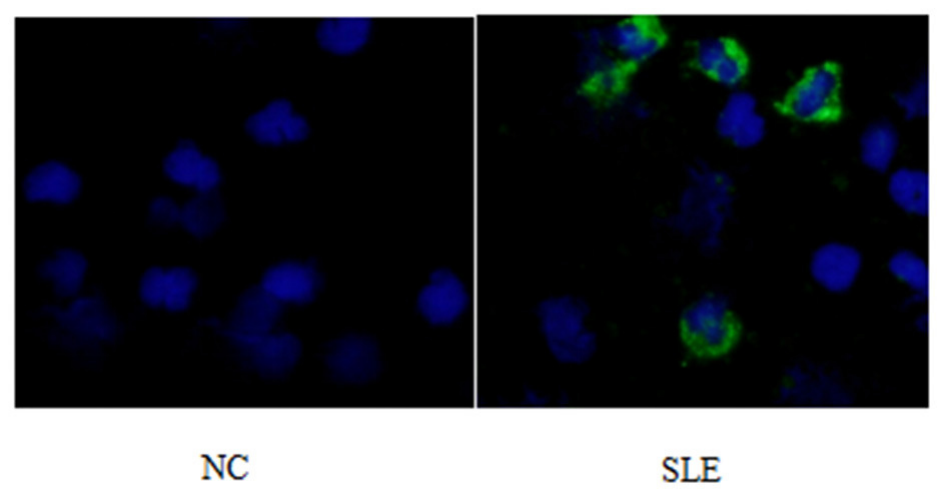
LC3 immunofluorescence staining results of peripheral blood lymphocytes in systemic lupus erythematosus group and normal children (NC) before treatment. Left, NC; Right, SLE.

**Figure 3:**
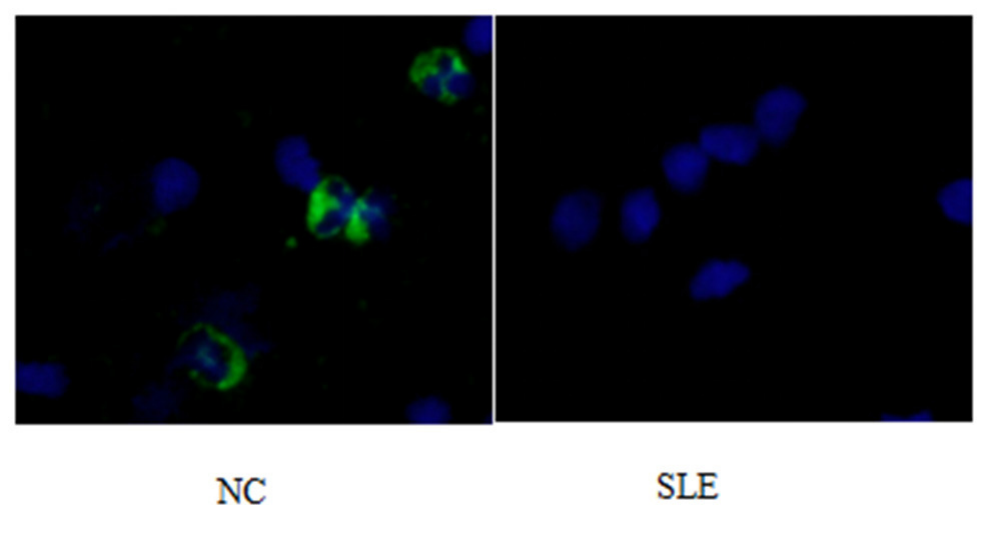
P62 immunofluorescence staining results of peripheral blood lymphocytes in systemic lupus erythematosus group and normal children (NC) before treatment. Left, NC; Right, SLE.

**Figure 4:**
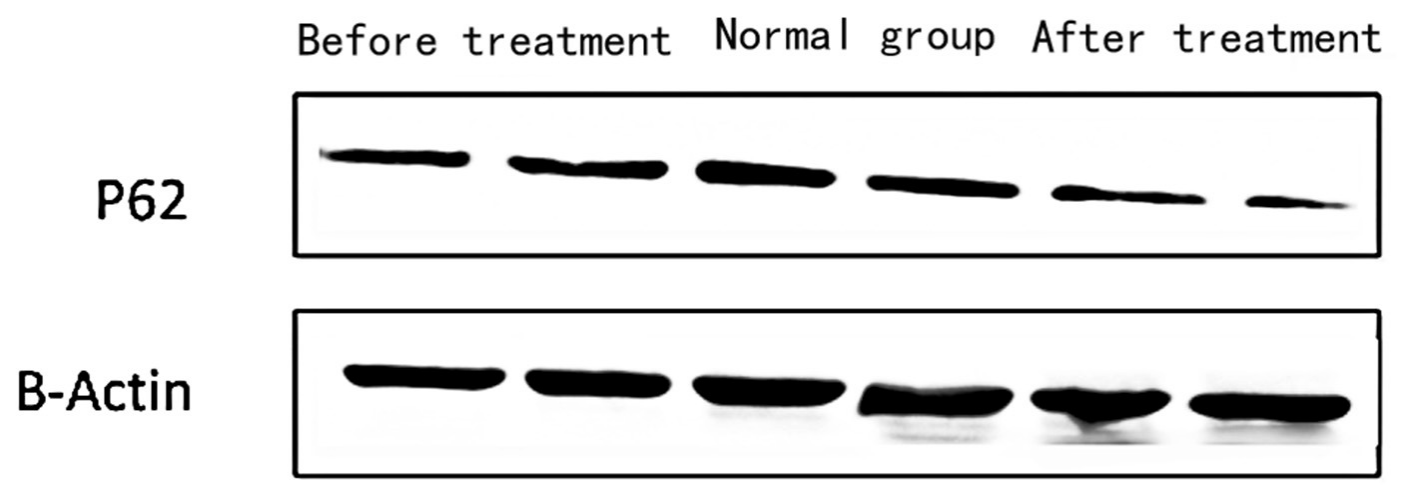
The expression of P62 in peripheral blood lymphocytes of children with SEL in the traditional treatment group at the end of 6 months and in the sirolimus group at the end of 6 months and before treatment. Two columns on the left: traditional treatment group; two columns on the middle: before treatment, two columns on the right: sirolimus group, β-Actin was used as the internal control for normalization.

**Figure 5:**
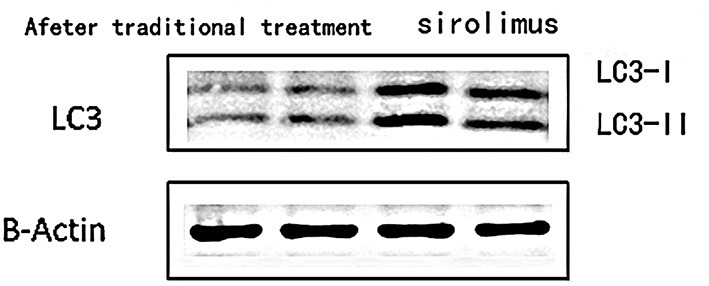
The expression of LC3 in peripheral blood lymphocytes in the traditional treatment group and sirolimus group at the end of 6 months. Two columns on the left: traditional treatment group; two columns on the right: sirolimus group, β-Actin was used as the internal control for normalization.

1) Compare the SLE group (Fifteen patients were randomly selected from the Traditional treatment group and the Sirolimus group and combined as the pre-treatment SLE group) before treatment with the normal children, the expression of LC3 protein in peripheral blood lymphocytes increased, with statistical significance ([0.3686 ± 0.0832] vs. [0.5175 ± 0.0721], *t* = −5.428, *P* = 0.0003); the expression of P62 protein in peripheral blood lymphocytes decreased, with statistical significance ([0.6005 ± 0.089] vs. [0.4965 ± 0.0731], *t* = 3.456, *P* = 0.001). Immunofluorescence was consistent with Western blotting results (see Figures 1–3 and Table 3).
2) Compare the SLE group before treatment with the traditional treatment group after 6 months of treatment, there was no significant difference in the expression of LC3 protein in peripheral blood lymphocytes, which was not statistically significant ([0.5175 ± 0.0721] vs. [0.5097 ± 0.0911], *t* = 0.445, *P* = 0.2). There was no significant difference in the expression of P62 protein in peripheral blood lymphocytes, which was not statistically significant ([0.4965 ± 0.0731] vs. [0.4942 ± 0.0688], *t* = 0.086, *P* = 0.4). Immunofluorescence was consistent with Western blotting results (see Figure 4 and Table 3).
3) Compare the sirolimus group with the traditional treatment group after 6 months of treatment, the sirolimus group showed increased expression of LC3 protein in peripheral blood lymphocytes, which was statistically significant ([0.6485 ± 0.1314] vs. [0.5097 ± 0.0911], *t* = −3.245, *P* = 0.003); the expression of P62 protein in peripheral blood lymphocytes decreased, with statistical significance ([0.4028 ± 0.0765] vs. [0.4942 ± 0.0688], *t* = 3.068, *P* = 0.005). Immunofluorescence was consistent with Western blotting results (see Figure 5 and Table 3).

**Table 3:** LC3 and P62 expression levels in peripheral blood lymphocytes of normal children (C), pre-treatment SEL (SLE), traditional treatment at the end of 6 months (A), sirolimus treatment at the end of 6 months (B) ($\bar{x}$ ± *s*).

**Group**	***n***	**LC3 relative expression**	**p62 relative expression**
Normal group	15	0.3686 686.0832	0.6005 005.0890
SLE	30	0.5175 175.0721	0.4965 965.0730
Traditional treatment group	28	0.5097 097.0911*	0.4942 942.0688*
Sirolimus group	28	0.6485 485.1314^*#^	0.4028 028.0765^*#^

Compared with SLE before treatment, *P < 0.05; Compared with the traditional treatment group, ^#^P < 0.05.

## Discussion

Abnormalities or regulatory disturbances in the process of autophagy are considered to be one of the main causes of abnormalities in the immune system of patients with systemic lupus erythematosus in recent years, and are closely related to the differentiation, proliferation and function of innate and adaptive immune cells. Obstruction of the autophagy process will lead to the untimely removal of dead cells, and the cleaning-up of DNA and RNA in the cells, and the long-term survival of T lymphocytes and B lymphocytes will be affected. As shown in Figures 1–3, compared with normal children, the level of autophagy in peripheral blood lymphocytes in children with systemic lupus erythematosus was significantly increased, as shown in Table 2 for quantitative analysis of autophagy-related proteins, and compared with normal children, the increase of peripheral blood lymphocyte autophagy-related protein expression in children with systemic lupus erythematosus has statistical significance, indicating that abnormal autophagy is involved in the pathogenesis of systemic lupus erythematosus.

In systemic lupus erythematosus, the inappropriate activation and presence of T lymphocytes and B lymphocytes are very important factors that lead to the infiltration of multiple organ inflammatory factors and the continuous production of autoantibodies. Animal model studies have found that the survival rate of B lymphocytes in mice with the autophagy gene ATG7 knocked out is reduced, and B lymphocytes cannot effectively differentiate into plasma cells, indicating that autophagy activation can promote the survival and normal function of B lymphocytes. In addition, the density of freshly isolated mitochondria in B-lymphocytes of mice with the autophagy gene ATG7 knocked out was significantly higher than that in the control group. This study showed that impairment of mitochondrial clearance leads to the reduced survival rate of B lymphocytes. On the contrary, by promoting autophagy, it can effectively clear mitochondria, improve the survival of B lymphocytes, and contribute to its normal function. T lymphocytes in patients with systemic lupus erythematosus also have many abnormalities, including abnormal activation of T lymphocytes and prolonged survival of T lymphocytes. In the T lymphocytes of patients with systemic lupus erythematosus, the mTOR pathway is activated. Activation of the mTOR pathway can lead to lupus activity and recurrence, and sirolimus as an effective mTOR pathway inhibitor can effectively reduce disease activity, as shown in Table 2, at the end of 3 and 6 months of treatment, the sirolimus group showed a more significant decrease in SLEDAI than the traditional treatment group, and the anti-dsDNA positive rate was lower. It can be seen that the PI3K/Akt/mTOR pathway is generally activated under the pathological condition of lupus, which promotes the abnormal activation of T lymphocytes to a certain extent. A recent study analyzed LC3 conversion rate as an indicator of T lymphocyte autophagy activity in mouse models of lupus and human lupus patients (14). Most current studies suggest that autophagy is more likely to occur in systemic lupus erythematosus. Increased levels of LC3 in cells reflect the formation or accumulation of autophagosomes. Studies have found that compared with healthy people, patients with systemic lupus erythematosus have significantly higher levels of lymphocyte autophagy. Clarke et al. (7) also found that in the lymphocytes of patients with systemic lupus erythematosus, the level of autophagy was significantly higher than that of the normal control, which was consistent with the results of this test. In this study, sirolimus was used to treat children with systemic lupus erythematosus at the end of June to collect peripheral blood extracted lymphocytes to detect the expression of autophagy-related proteins. After treatment with sirolimus, the level of lymphocyte autophagy is greater than that in children with systemic lupus erythematosus than in healthy children and greater than that in the traditional treatment group. Combined with the clinical indicators of children in the same period as shown in Table 2, the SLEDAI decrease in the sirolimus treatment group at the end of June was significantly higher than that in the traditional treatment group, and the percentage of lymphocytes showed an increase compared to the traditional treatment group. As shown in Table 2 the complement C3 and complement C4 shown in the two groups of children showed an increase, but there was no significant difference, and further analysis was needed for long-term observation. The increased autophagy level after sirolimus treatment may be due to increased autophagy induction or blocked autophagy pathway, but at this time the increase in autophagy level may just reset the autophagy disorder so that the homeostasis of the cell is re-established, as a result, patients recover from the disease. Comprehensive analysis of the research results and general conclusions of previous scholars at home and abroad leads to questions of whether the mTOR signaling pathway in lymphocytes of patients with systemic lupus erythematosus is not mainly involved in regulating the level of autophagy in lymphocytes, whether there is a regulatory defect in the mTOR signaling pathway in lymphocytes of patients with systemic lupus erythematosus, and whether there are more important signaling pathways involved in the regulation of lymphocyte autophagy in patients with systemic lupus erythematosus? The specific mechanism needs to be further studied.

There is also an in-depth study on the changes of peripheral blood T and B lymphocyte autophagy levels in patients with systemic lupus erythematosus before and after treatment with hormones and immunosuppressive agents. It was found that the autophagy level of T lymphocytes increased at the time of onset and decreased after treatment, and the level of autophagy was positively correlated with the SLEDAI score, while the level of autophagy of B lymphocytes decreased at the time of onset, and recovered after treatment, and was positively correlated with the complement level, and there was no correlation with SLEDAI or serum Anti-dsDNA antibody titers (15). As shown in Table 2, the sirolimus treatment group is more effective than the traditional treatment group, but the autophagy level is not related to the clinical complement level, SLEDAI score, and Anti-dsDNA antibody level. It may be because peripheral blood mononuclear cells are known to contain multiple cell types, but this study did not further extract and classify lymphocytes, study the autophagy level of each type of cell separately, and how the autophagy level changes after treatment. These are the shortcomings of this test, which may lead to deviations in the results and need to be improved in future research.

The mechanism of autophagy involved in the pathogenesis of systemic lupus erythematosus also includes defects in the process of autophagy, which may cause the DNA and RNA in damaged or dead cells to be effectively degraded and reused, thus providing a permanent source of antigen. Pathological processes such as excessive production of type interferon (IFN) and the clearance obstacles of circulating immune complexes may involve autophagy defects. Theoretically, there may be a lot of invalid autophagy in the increase of autophagy level under pathological conditions. It is speculated that invalid autophagy can lead to the accumulation of damaged mitochondria and damaged or dead cells. Whether directly or after the formation of antigen-antibody complexes, a large number of DNA/RNA protein complexes of damaged or dead cells are involved in the destruction of self-tolerance and the initiation of autoimmune reactions, activating the innate immune system, which leads to the production of inflammatory cytokines. Therefore, after using sirolimus, the enhanced autophagy level measured in the test is shown in Figures 4, 5, which may enhance the effective autophagy level, effectively remove damaged mitochondria and damaged or dead cells, effectively suppress the source of self-antigens, and reduce the production of autoantibodies and the production of inflammatory cytokines.

Although there has been a lot of research at home and abroad, the mechanism that causes the systemic lupus erythematosus to reduce its innate immune autophagy activity and increase the lymphocyte autophagy activity is still unclear, but the presence of disorder in the signal pathways involved in the regulation of lymphocyte proliferation, differentiation has been c determined to exist. The earliest guess and research about the involvement of autophagy in the pathogenesis of systemic lupus erythematosus is based on animal models and clinical treatments. Drugs for treating systemic lupus erythematosus, such as glucocorticoids, hydroxychloroquine, and sirolimus, all belong to mTOR inhibitors, which have beneficial effects. The comprehensive analysis found that the treatment of systemic lupus erythematosus focuses on resetting the flux of autophagy and keeping autophagy in a balanced state to regulate the growth and differentiation of body cells, so as to achieve the purpose of maintaining cell homeostasis.

In this experiment, we combined the clinical laboratory results to observe the efficacy of sirolimus in the treatment of systemic lupus erythematosus. For the 28 patients with systemic lupus erythematosus, after treatment with sirolimus, the percentage of lymphocytes increased, SLEDAI decreased, Anti-dsDNA positive rate decreased statistically, compared with the traditional treatment group. Complement levels increased, but there was no difference from the results of the traditional treatment group. Dyslipidemia is a common complication of GC treatment in children with systemic lupus erythematosus. As shown in Table 2, the changes in triglyceride and total cholesterol levels were observed. The application of sirolimus did not aggravate the occurrence of dyslipidemia. However, one child in the sirolimus group developed transient nausea with abdominal pain on the 2nd day after oral administration, symptom was mild, spontaneous remission did not recur, and the child was not withdrawn from the study.

This study also has some limitations. First of all, due to the small sample size, possible result bias and short observation time, the effect of mTOR inhibitors on the long-term prognosis of SLE was not observed in this study. Secondly, limited time to observe the clinical efficacy and side effects of the drug, and the lack of double-blind randomized controlled studies, which is the deficiency of this experiment. In the future, we will need more molecular biology, genomics, animal model tests, etc. to explore in detail the study of autophagy and its related pathways in the pathogenesis of systemic lupus erythematosus, as well as a larger sample of clinical trials to observe the efficacy and side effects of Sirolimus.

In conclusion, the pathogenesis of systemic lupus erythematosus is related to abnormal levels of peripheral blood lymphocyte autophagy. Compared with traditional treatment, sirolimus can increase the level of autophagy in peripheral blood lymphocytes, indicating that it may play a role in the treatment of systemic lupus erythematosus by regulating the level of autophagy. Compared with traditional treatment, sirolimus has faster recovery of clinical laboratory indicators, higher recovery percentage, and more significant effect.

## Data Availability Statement

The raw data supporting the conclusions of this article will be made available by the authors, without undue reservation.

## Ethics Statement

The studies involving human participants were reviewed and approved by the Ethics Committee of Second Hospital of HeBei Medical University. Written informed consent to participate in this study was provided by the participants' legal guardian/next of kin.

## Author Contributions

XW and QS conceived of the study. ZD, XZ, and NZ participated in its design and coordination. BY, YW, and ZR helped to draft the manuscript. All authors read and approved the final manuscript.

## Conflict of Interest

The authors declare that the research was conducted in the absence of any commercial or financial relationships that could be construed as a potential conflict of interest.

## Publisher's Note

All claims expressed in this article are solely those of the authors and do not necessarily represent those of their affiliated organizations, or those of the publisher, the editors and the reviewers. Any product that may be evaluated in this article, or claim that may be made by its manufacturer, is not guaranteed or endorsed by the publisher.
